# Repurposing Regulatory Rodent Toxicology Safety Archives to Identify Candidate Hormetic Longevity Agents Highlights Emergent Mitochondrial Mechanisms

**DOI:** 10.1111/acel.70797

**Published:** 2026-11-01

**Authors:** Satomi Miwa, Olena Kucheryavenko, Kristin Herrmann, Adelaide Raimundo, Feng Xue, Karthik Mohanraj, Christopher Saunter, Lars Niemann, David Weinkove, Thomas von Zglinicki

**Affiliations:** ^1^ Biosciences Institute, Faculty of Medical Sciences Newcastle University Newcastle upon Tyne UK; ^2^ German Federal Institute for Risk Assessment (BfR) Berlin Germany; ^3^ Leipzig University Leipzig Germany; ^4^ Department of Biosciences Durham University Durham UK; ^5^ Magnitude Biosciences Limited, NETPark Plexus Durham UK

**Keywords:** ageing, *Caenorhabditis elegans*, hormesis, lifespan, mitochondria, mitochondrial uncoupler, pesticides, toxicology

## Abstract

Efforts to extend longevity and healthspan have benefited from the use of in vitro systems, short‐lived model organisms and computational approaches, but translation to humans depends on demonstrating efficacy and safety in mammalian in vivo models. However, rigorously controlled rodent lifespan studies are slow, costly and low‐throughput. Hormesis, the induction of adaptive stress response pathways by low‐level toxic or metabolic challenges, provides a mechanistic means for improved resilience, but testing hormetic dose–response relationships in mammals is logistically challenging. Regulatory long‐term toxicity studies, mandated for chemical authorisation, routinely generate lifespan and chronic toxicity data from large cohorts across multiple dose groups and therefore represent an underused resource for detecting unexpected survival benefits. We mined a random subset of the German Federal Institute for Risk Assessment (BfR) electronic archive for long‐term rodent pesticide studies in ≥ 50 animals per dose and sex. From qualifying studies with 154 substances, we identified 27 substances (18% of entries) that significantly increased survival at study termination (≥ 10%, *p* < 0.05). Fifteen publicly disclosable substances were examined further; roughly half perturbed mitochondrial oxygen consumption rates in mammalian cells, and bromoxynil acted as a mitochondrial uncoupler. Back‐translation to *Caenorhabditis elegans* showed bromoxynil improved multiple healthspan indicators without compromising organismal health. Regulatory toxicity archives can thus be repurposed to identify candidate compounds, targets and mechanisms for mammalian healthy ageing research, complement conventional screening pipelines, align with 3Rs by maximising existing data, and supply high‐quality datasets for AI‐driven prioritisation of interventions for rigorous preclinical follow‐up.

## Graphical abstract

Mining regulatory rodent toxicology archives for pesticides uncovers substances that paradoxically enhance survival, revealing an underused resource for longevity discovery. Many candidates act via mild mitochondrial perturbations, providing a foundation to investigate stress‐response mechanisms, dose–response trade‐offs and therapeutic modalities for healthy ageing interventions.

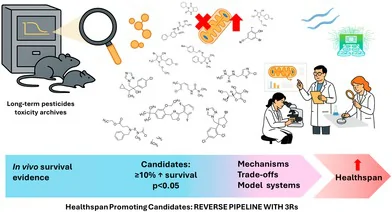

1

Efforts to extend longevity and healthspan are intensifying as populations confront rising burdens of chronic, age‐related disease. Discovery pipelines span in vitro systems, short‐lived model organisms and computational approaches, but translation to humans still requires demonstration of efficacy and safety in mammalian in vivo models. Large, rigorously controlled lifespan studies in genetically heterogeneous mice remain the preclinical gold standard but are slow and costly, which constrains throughput and limits the number of interventions tested at scale. The NIA Interventions Testing Program has provided a standardised framework for multi‐site testing since 2004 (Nadon et al. 2017), yet reproducible extensions of mouse lifespan remain uncommon, and are typically modest and sex‐specific.

Hormesis, the adaptive activation of multiple protective stress‐response pathways by low‐level toxic or metabolic challenges, offers a mechanistic rationale for many longevity interventions. Numerous lifespan‐ and healthspan‐extending regimens, including dietary restriction and several pharmacological agents, are thought to operate at least in part through hormetic mechanisms that increase stress resistance and physiological resilience. Hormesis is well documented in short‐lived models such as *Caenorhabditis elegans* (Edwards et al. 2013; Hunt et al. 2011) and *Drosophila* (Schriner et al. 2013), where low‐dose stressors and mild mitochondrial perturbations frequently produce robust increases in lifespan and stress tolerance. Demonstrating hormesis in mammals is substantially more challenging because it requires long‐term dose–response studies with large cohorts and lifespan as an endpoint.

Regulatory long‐term toxicity studies submitted to national and international agencies constitute an underused repository of high‐quality rodent survival data. These studies routinely test multiple dose groups in large cohorts under stringent quality‐control frameworks, including chronic toxicity and carcinogenicity evaluations, and use survival as a primary endpoint (Niemann et al. 2023). Archives maintained by bodies such as the European Food Safety Authority (EFSA) and the US Environmental Protection Agency (EPA) thus contain resources, for which secondary analysis may reveal unexpected survival benefits. Mining these archives can provide an opportunity to identify compounds that paradoxically increase survival at study termination and to infer shared mechanistic features that could be harnessed for healthy ageing.

To explore this possibility, we mined the electronic Archive of Studies and Assessments of the German Federal Institute for Risk Assessment (BfR), which contains information about plant protection and biocidal products, their active substances and co‐formulants, for long‐term rodent studies of pesticide active substances tested at multiple doses (typically three) using at least 50 rats and mice per dose and sex.

Typically, studies in rats lasted over 2 years, yielding median survival at endpoint in controls of 52% in males and 48% in females. In mice, only 33% of studies were conducted over 22 months, with similar median survival (57% in males and 52% in females); the remaining studies were shorter, resulting in higher survival, hence reduced statistical power to detect survival changes.

In a randomly taken 545 database entries (Figure S1), 154 contained long‐term toxicity and survival data. Among these, 27 substances (18%) increased survival (at least 10%) at study termination (*p* < 0.05) in at least one species, sex, or dose. Only one, ascorbic acid, had previously been shown to extend lifespan in male mice (Massie et al. 1984). Most of them also exhibited non‐significant survival increases (10% or more) across other concentrations and cohorts, with survival decreases being rare (Table 1).

**TABLE 1:** 15 substances that significantly changed rodent survival at study termination.

Substance	Dose range (ppm)	Duration (weeks)	Males			Females		
			Survival (species: dose)	FI	BW	Survival (species: dose)	FI	BW
Alanycarb (ALAN)	R: 30–300 M: 100–1000	R: 104 M: 84	R: 30 M: 100 M: 300*			R: 300 M: 100 M: 300 M: 1000		
Ascorbic acid (ASC)	25,000, 50,000	R: 105 M: 105	R: 50,000 M: 25,000 M: 50,000**					
Bifenazate (BIF)	R: 20–200/160 (m/f) M: 10–225	R: 104 M: 78	R: 80 R: 200* M: 100* M: 225	↓	↓ ↓ ↓			
Bromoxynil (BROMOXY)	R: 60–600 M: 10–100	R: 104 M: 78	R: 60 R: 600 M: 30		↓	R: 60 R: 190** R: 600*** M: 10 M: 30 M: 100		↓ ↓
Bromuconazole (BROMUC)	R: 20–2000 M: 20–500	R: 104 M: 78	R: 1000 R: 2000 M: 100* M: 500			M: 100 M: 500		
Chlorfenapyr (CHLORFE)	R: 60–600 M: 20–240	R: 104 M: 78				R: 600* M: 240*	↓ ↓	↓ ↓
Chlortoluron (CHLORTO)	R: 100–2500 M: 100–2500	R: 115 M: 105	R: 100* M: 100* M: 500			R: 100* R: 500* R: 2500*	↓	↓
Clothianidin (CLOTH)	R: 150–3000 M: 100–2000/1800 (m/f)	R: 104/103 (m/f) M: 78	R: 1500 R: 3000**** M: 100 M: 1250	↓	↓ ↓	R: 1500 R: 3000*** M: 350 M: 1800*	↓ ↓ ↓	↓ ↓ ↓
Cyantraniliprole (CYANT)	R: 20–20,000 M: 20–7000	R: 104 M: 79	R: 20 M: 20* M: 150*			R: 20 R: 2000 M: 7000	↑	
Cyproconazole (CYPROCON)	R: 20–350 M: 5–200	R: 121 M: 81/88 (m/f)	R: 350 M: 15* M: 100* M: 200**		↓ ↓	M: 5 M: 100*** M: 200		↓ ↓
Cyprodinil (CYPRODIN)	R: 5–2000 M: 10–5000	R: 104 M: 78	M: 5000*		↓	R: 75 M: 5000		↓
Dimethenamid (DIMET)	R: 100–1500 M: 30–3000	R: 104 M: 94	R: 700 R: 1500* M: 30 M: 300 M: 3000		↓	R: 1500 M: 30 M: 300 M: 3000		↓
Etoxazole (ETOX)	R: 50–10,000 M: 2250,4500	R: 104 M: 78	R: 10,000 M: 2250	?	↓	R: 10,000*	↓	↓
Fenpyroximate (FEN)	R: 10–150 M: 25–800	R: 104 M: 78	R: 25 R: 75 R: 150 M: 400	↓ ↓	↓ ↓	R: 25 R: 75* R: 150 M: 100	↓ ↓	↓ ↓ ↓
Lenacil (LEN)	R: 250–25,000 M: 100–7000	R: 104 M: 78	R: 2500* R: 25,000 M: 2500		↓	R: 250 R: 25,000 M: 100 M: 7000		↓

*Note:* All doses (ppm in food) resulting in > 10% change in survival at study termination are shown for rats (R) and mice (M). Values in black indicate increased survival, and values in red indicate decreased survival. Statistical significance is indicated as follows: *p < 0.05, **p < 0.01, ***p < 0.005, ****p < 0.001, based on Kaplan–Meier analysis, Cox proportional hazards regression, or trend tests with appropriate pairwise comparisons. In addition, results that remain significant after Benjamini–Hochberg false discovery rate (FDR) correction (*q* ≤ 0.20) are shown in bold. Changes in food intake (FI) and body weight (BW) are indicated by arrows where differences from controls exceeded 10% over the majority of the study period.

Out of these 27 substances, 15 can be publicly disclosed for further investigation. Four (alanycarb, ascorbic acid, bifenazate, bromuconazole) increased survival exclusively in males, while two (chlorfenapyr, chlortoluron) did so only in females. Sex‐biases for the remaining nine substances were also observed but were typically concentration and/or species‐dependent (Table 1). Figure S2 provides examples of survival curves for bromoxynil. Bonferroni and Benjamini‐Hochberg‐Analyses indicated that the most robust survival effects remain significant under stringent correction, while others fall below formal FDR thresholds (Table S1).

Chlorfenapyr, chlothianidin and etoxazole tended to reduce food intake and body weight at the doses that improved survival (Table 1), suggesting that a ‘dietary restriction effect’ might have played a role. In contrast, bromoxynil, cyproconazole, cyprodinil and lenacil reduced body weight without reducing food intake, albeit sex‐specifically, implying they could have acted as ‘exercise mimetics’ or produced organ toxicity.

The most common chronic toxic effect, typically at the maximum dose tested, was increased liver weight, often with centrilobular hypertrophy, indicating a detoxification response. No substance was regarded as genotoxic. Indications for reproductive toxicity, neurotoxicity, carcinogenicity and further chronic toxicity are summarised in Table S2. A clear safety margin between beneficial and toxic doses would thus be essential. What are the mechanisms by which these substances promote survival in mammals? In the literature, their effects on electron transfer systems were frequently reported, although mostly in non‐mammalian systems: inhibition of the electron transport chain of the Photosystem II in plants (bromoxynil, chlortoluron, lenacil) and of plant or insect mitochondria (bifenazate, chlorphenapyr, fenpyroximate) (Table S3). Given perturbations of mitochondria can activate stress response pathways and extend lifespan in *C. elegans* and mice (Pulliam et al. 2014; Cheng et al. 2023), the effects of these 15 substances on cellular oxygen consumption rates (OCR) in mammalian cells were examined at both low (LD20) and high (LD80) doses (Figure S3) for each substance, respectively, using a Seahorse XF Analyser.

In intact cells with high doses (LD80), multiple substances significantly inhibited cellular OCR, whereas bromoxynil and chlorfenapyr stimulated it. At LD20, only bifenazate and fenpyroximate inhibited and bromoxynil stimulated it (Figure 1A,B). To examine mitochondria‐specific effects, OCR was measured in permeabilised cells using complex‐specific substrates. Complex I‐specific inhibition was observed for chlortoluron, cyprodinil, bromuconazole and bifenazate, and for sites other than complex I by etoxazole and fenpyroxymate (Figure S4). Bromoxynil and chlorfenapyr stimulated mitochondrial OCR during active respiration at state 3 OCR further. Mitochondrial uncoupler activity of bromoxynil was confirmed in state 4 OCR (in the presence of oligomycin, Figure S5), which is in line with the in vivo observation showing body weight reduction without changes in food intake (Table 1). Chlorfenapyr is a pro‐drug to the active uncoupler, AC 303,268 (tralopyril) which typically requires activation by cytosolic oxidases (Krieger 2001) explaining that no mitochondrial uncoupling activity was observed in permeabilised cells (Figure S5). Collectively, the results suggest that perturbation of mitochondrial function (summarised in Table S2) is a frequent mechanism in inducing pro‐survival stress response pathways among the 15 substances which promoted survival in mammals.

**FIGURE 1:**
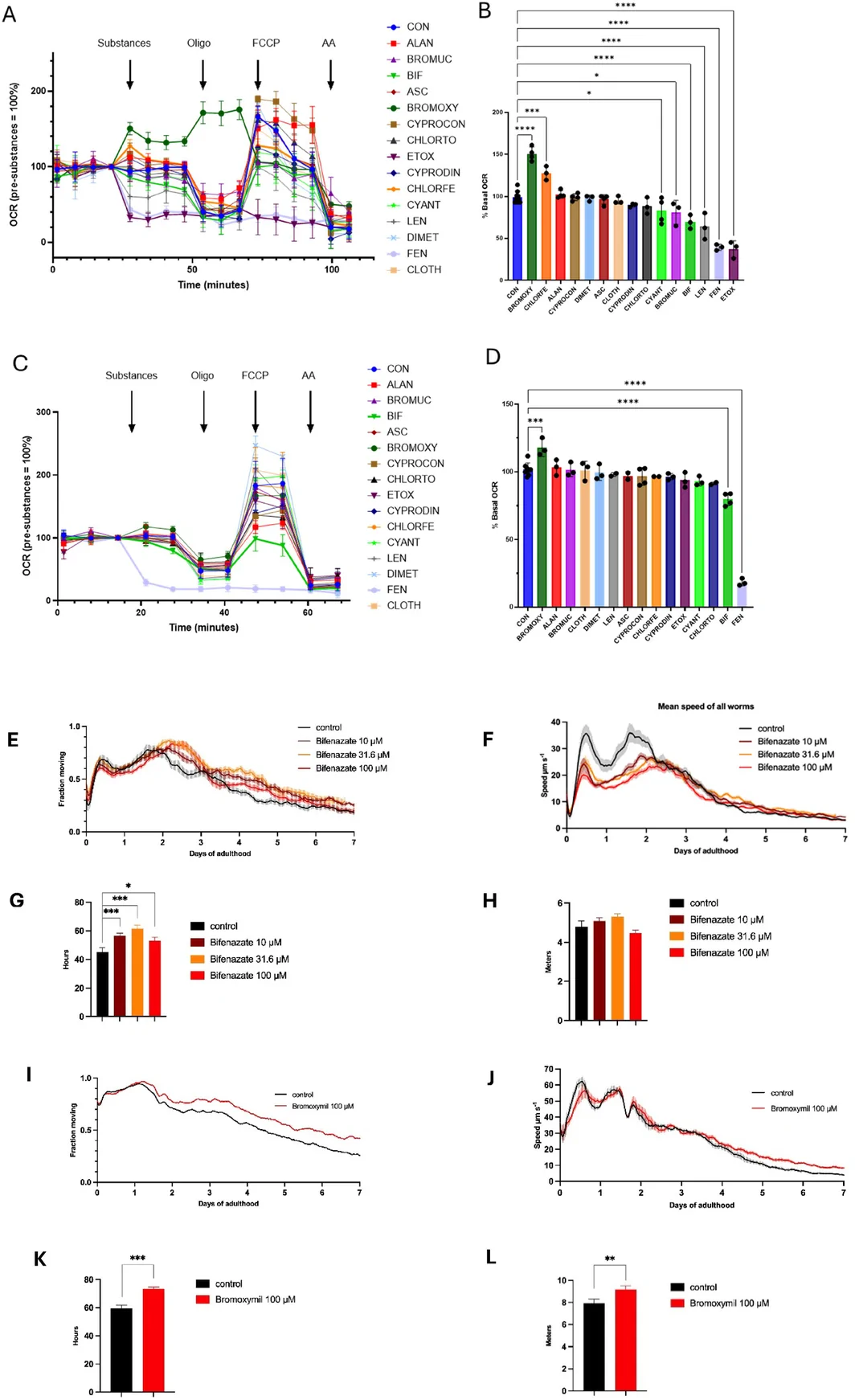
(A–D) Effects of 15 substances at LD80 (A, B) and LD20 (C, D) on intact cell oxygen consumption rates (OCR) in human fibroblasts. (A, C) Time course measurements of OCR. Sequential measurements of OCR at basal, substances at LD80 (A) or LD20 (C) concentrations, with oligomycin (Oligo, 0.5 μM), with uncoupler FCCP (2.5 μM) and with Antimycin A (AA, 2.5 μM) using a Seahorse XF Analyser. Data integrated from 3 to 9 (LD80) or 3–8 (LD20) measurements in 2–3 independent experiments. (B, D) % OCR in the presence of substances at LD80 (B) or LD20 (D) relative to that in the absence. One way ANOVA, differences to controls (CON) indicated by asterisks with *p < 0.01, ***p < 0.001, ****p < 0.0001. Full names for abbreviated substance names are indicated in Table 1. (E–L) Effects of bifenazate (E–H) and bromoxynil (I–L) on healthspan indicators in *C. elegans* . (E, I) Fraction of moving worms over their life history. (F, J) Mean movement speed of all worms over time. Smoothed averages (bold lines) ± SEM (shaded areas), *n* ≥ 350 animals per condition. (G, K) Mean hours all worms moved between Days 2 and 7. (H, L) Mean distance travelled by worms between Days 2 and 7. Means ± SEM over all worms from Day 2 to 7, differences to controls indicated by asterisks with *p < 0.05, **p < 0.02, ***p < 0.001.

Could these substances extend not only organismal lifespan but also healthspan? While anti‐ageing interventions on lifespan effects in short‐lived organisms may not always be replicated in mammalian models, healthspan outcomes have been suggested to have translational values (Bene and Salmon 2023). Accordingly, *C. elegans* healthspan was examined using two reliable parameters on spontaneous motility (Weinkove and Zavagno 2021; Zavagno et al. 2024), chosen as sensitive readouts of neuromuscular and metabolic function rather than proxies for organismal survival: the proportion of moving worms and their average movement speed. Bifenazate and bromoxynil were selected as proof‐of‐concept examples, with the former inhibiting and the latter uncoupling mitochondria, both at LD20.

Using the highest concentrations that did not result in acute or developmental toxicity, bifenazate increased the fraction of moving worms at higher ages, with an optimal effect at the intermediate concentration of 31.6 μM (Figure 1E) but did not increase their movement speed, including a reduction in younger animals (Figure 1F). Total time spent moving increased (Figure 1G), although distance travelled did not (Figure 1H), together indicating that bifenazate alters movement patterns, producing slower but more sustained activity; lifespan, however, was not significantly increased (Figure S6). Bromoxynil at 100 μM resulted in more worms remaining active during the first 7 days of adulthood (Figure 1I), faster movement at later ages (Figure 1J) and increased total movement time (Figure 1K) and distance (Figure 1L), indicating improved functional healthspan in *C. elegans* . Despite these functional improvements, lifespan was decreased (Figure S6), suggesting that the hormetic window may change or narrow with age, whereby mitochondrial uncoupling stress may shift from beneficial to detrimental as animals age. Finally, consistent with hormesis, both substances activated oxidative stress responses: survival upon paraquat exposure increased for each agent (Figure S7), and bromoxynil additionally induced *gst‐4* expression, consistent with enhanced detoxification. The absence of obvious *hsp‐6* upregulation (Sup. Experimental Procedures) aligns with bromoxynil acting through bioenergetic stress rather than proteostasis‐related signalling.

In summary, data mining has found 27 substances (18% among sampled entries) associated with increased survival at study termination (*p* < 0.05). Among disclosed substances, roughly half perturb mitochondrial function, implicating mitochondrial stress responses as strong candidate mechanisms supporting enhanced survival in mammals. One such agent, the mitochondrial uncoupler bromoxynil, improved healthspan in *C. elegans* , indicating that organismal functional metrics can be favourably modulated independently of lifespan and through hormesis‐like effects.

These findings demonstrate that long‐term toxicity archives, such as those submitted to national and international regulatory agencies, can be repurposed to identify candidate compounds, targets and mechanisms relevant to mammalian healthy ageing. Leveraging such datasets complements conventional screening pipelines, aligns with the 3Rs by maximising information from existing studies, and provides rich training material for AI‐ and *in silico*–driven drug‐discovery approaches that require large, high‐quality datasets to prioritise interventions for rigorous follow‐up in mammalian models.

## Author Contributions

T.vZ., S.M., L.N. and D.W. designed and supervised the study. O.K. and K.H. analysed the toxicology data under supervision by L.N. S.M. performed and analysed mammalian cell experiments. C.S., A.R., F.X. and K.M. designed, executed and analysed the *C. elegans* experiments. All authors involved in drafting and revising the manuscript have given final approval for publication and agreed to be accountable for all aspects of the work.

## Funding

This work is supported by EU FP7‐PEOPLE PITN‐GA‐2012‐316964 (MARRIAGE) and H2020 WIDESPREAD Project 857524 (MIA Portugal) to TvZ, and the Werner Baltes Fellowship, BfR to OK.

## Disclosure

Disclaimer: This publication has been approved by the German Federal Institute for Risk Assessment; however, the opinions expressed in the publication do not represent the regulatory position of the institute.

## Conflicts of Interest

T.vz., O.K., L.N., K.H., A.R., F.X., K.M. and S.M. have nothing to declare. D.W. and C.S. are shareholders of Magnitude Biosciences Limited.

## Supporting information


**Table S1:** Multiple testing correction of survival comparisons using Bonferroni and Benjamini–Hochberg (BH) procedures.
**Table S2:** Toxicity summary for 15 substances that improve rodent survival in long‐term studies.
**Table S3:** Possible mechanisms of action of 15 substances as per literature and summary of their mitochondrial effects in human fibroblasts.
**Figure S1:** Screening strategy.
**Figure S2:** Example survival curves for rats and mice treated with bromoxynil.
**Figure S3:** Human lung fibroblasts (MRC5) viability following 48 h treatment with 15 selected substances at the indicated concentrations.
**Figure S4:** Effects of 15 substances on complex I‐ and II‐ linked mitochondrial oxygen consumption rates (OCR) in permeabilised human lung fibroblasts.
**Figure S5:** Mitochondrial OCR (at state 4) in permeabilised MRC5 cells, supplemented with complex I‐linked substrates 10 mM Pyruvate and 1 mM Malate in the presence of 5 μM oligomycin.
**Figure S6:** *C. elegans* lifespan under bromoxynil or bifenazate treatments.
**Figure S7:** Response of *gst‐4* to bifenazate and bromoxynil and protection from paraquat.

## Data Availability

Survival and toxicology data are in the Assessment Reports for the individual active substances of plant protection products, available from the BfR or the EFSA upon request.
