# Supplementary material for: Repurposing Regulatory Rodent Toxicology Safety Archives to Identify Candidate Hormetic Longevity Agents Highlights Emergent Mitochondrial Mechanisms
Source: Aging Cell. 2026 Nov 1;25(11):e70797. doi: 10.1111/acel.70797 (PMC13724199; doi:10.1111/acel.70797)
Supplement: Supplementary file 1 [file ACEL-25-e70797-s001.docx]

**Supplementary Material:**

**Experimental Procedures:**

*Database screening*

The Federal Institute for Risk Assessment (Bundesinstitut fuer Risikobewertung BfR, https://www.bfr.bund.de/) is responsible for risk assessment of pesticides, plant protection and biocidal products in Germany. Its electronic Archive of Studies and Assessments contains more than 1000 substance entries, comprising plant protection products and biocidal products, their active ingredients, and co-formulants. Comprehensive data on toxicology, including, importantly for the present study, long-term survival data in males and females of two species (typically rats and mice) are usually available for the active substances. Automated electronic retrieval of survival data was not possible; therefore all survival data were manually retrieved. Quality of data was checked by a second independent observer. The screening strategy is shown in Fig. S1. Long-term survival data were available for 154 out of 545 randomly selected substances, with cohorts typically comprising 50 - 60 animals per species, sex, and substance concentration (between three and five concentrations per substance), resulting in over 2000 individual survival comparisons. As expected, the majority of these comparisons showed no increase, or a decrease, in survival.

To identify candidates with potential pro-survival effects, substances that met both of the following criteria were selected: (i) at least a 10% increase in survival relative to controls in at least one strain, sex or concentration; and (ii) a corresponding statistical significance of p ≤ 0.05 (suppl. Fig. S1). This approach identified 27 candidate substances, of which 15 could be publicly disclosed (Tab. 1), yielding survival data from 168 species-sex-substance-concentration cohorts.

Survival analyses were performed by Kaplan-Meier product-limit method, followed by Gehan-Breslow test with Holm-Sidak pairwise comparisons or Cox proportional hazards regression, as appropriate. To provide transparency regarding multiple testing, the 10 most significant survival differences at 10% and 20% false discovery rates were additionally identified using both Bonferroni and Benjamini–Hochberg corrections and are reported in Supplementary Table S1.

As this was designed as an exploratory, hypothesis-generating analysis, the p<0.05 threshold without formal correction of multiple comparisons was retained for candidate identification.

*Cell viability assay*

Human lung fibroblasts (MRC5) were grown in a controlled environment of 5% CO_2_ and ambient oxygen at 37 °C in Dulbecco’s Modified Eagle’s Medium (DMEM; Sigma Aldrich, D5671), supplemented with 10% heat-inactivated fetal bovine serum (FBS; Sigma Aldrich, F9665), 100 U/ml penicillin and 100 mg/ml streptomycin (Sigma Aldrich, P4333), and 2 mM L-Glutamine (Sigma Aldrich, G7513). Cells were seeded on a 96-well plate (15,000 cells per well), and were treated with each of the 15 substances for 48 hours at concentrations ranging from 0.1μM to 5mM, except clothianidin, cyantraniliprol, dimethenamid, fenpyroximate and lenacil, where 50µM was the highest concentration due to their poor solubility, and this concentration was used as ‘pseudo LD80 concentration’ unless LD80 was not reached at lower concentrations. Cell viability was assessed using CellTiter-Glo® Luminescent Cell Viability Assay (Promega) according to manufacturer’s instructions.

*Oxygen consumption rates (OCR) measurements*

Intact cell oxygen consumption rates (OCR) were measured using human lung fibroblasts (MRC5) in a Seahorse XF24 Analyzer (Agilent Technologies) in unbuffered Dulbecco's Modified Eagle Medium supplemented with 5mM glucose, 2mM L‐glutamine and 3% FBS. The following compounds were added sequentially to test the effects of each of the 15 substances on cellular OCR: 0.5μM oligomycin to inhibit ATP synthase (hence inhibiting mitochondrial ATP generation), 2.5μM carbonyl cyanide p‐trifluoromethoxy‐phenylhydrazone (FCCP) to stimulate OCR, and 2.5μM Antimycin A to inhibit complex III.

To examine mitochondria-specific effects of the 15 substances, mitochondrial OCR was assessed by permeabilizing the cells with XF Plasma Membrane Permeabilizer (Agilent Technologies) according to the manufacturer’s instructions. Initially, complex I-linked substrate, pyruvate (10mM) and malate (1mM) were supplemented and state 3 OCR was achieved by the addition of 4mM ADP. Each of the 15 substances was then added to examine its complex I specific effect; state 3 OCR was chosen to maximise the sensitivity to detect any inhibitory effects of the substances. Rotenone (5μM) was added next to inhibit complex I, followed by the complex II-linked substrate succinate (4mM) to test the effects of each of the substances on other sites than complex I. To specifically test mitochondrial uncoupling function of bromoxynil and chlorfenapyr, permeabilised cell experiments were conducted at state 4 condition (in the presence of oligomycin; using complex I-linked substrate), along known uncouplers, FCCP, niclosamide and BAM15 as positive controls.

*C. elegans healthspan determination*

To examine the effects of candidate substances that perturb mitochondrial function on organismal healthspan in a complementary *in vivo* model, two lead substances were selected based on effect size, reproducibility across cohorts, absence of concomitant reductions in food intake, and evidence of mitochondrial perturbation at low doses. This prioritisation strategy identified bromoxynil and bifenazate as representative substances with distinct mitochondrial effects (uncoupling versus inhibition), enabling assessment of their functional consequences at the organismal level.

First, bromoxynil and bifenazate were tested for potential effects on OP50 bacterial growth starting at the highest concentrations that were soluble in DMSO. Bromoxynil suppressed bacterial growth at higher concentrations (≥500 µM), but not at the lower concentrations (≤100 µM) used for *C. elegans* healthspan and lifespan assays, whereas bifenazate showed no detectable effects on bacterial growth up to the highest concentration tested (158 µM). While no visible effects on bacterial abundance were observed under experimental conditions, potential effects on bacterial metabolism cannot be excluded and are acknowledged as a limitation of the test system.

Worms to be tested (SS104 *glp-4(bn2)* obtained from the *Caenorhabitis* Genetics Center, University of Minnesota) were maintained at 15°C on defined medium and *E. coli* OP50 (1). For healthspan testing, 3 days after a timed egg lay worms were shifted to 24°C. The following day worms at the L4 stage were transferred onto defined medium agar Petri dishes containing the substances to be tested and DMSO solvent controls. Concentrations of the substances were chosen as the highest tested that had no effect on *C. elegans* fertility or development and subsequent dilutions. All plates contained 1% DMSO. Movement of the population of worms was monitored from the L4 stage onwards using the WormGazer^TM^ technology as described (1). Outputs were the fraction of the population moving above a threshold speed and the mean speed of those worms. The mean speed of all worms was obtained by multiplying those parameters together. Statistical analysis was done as described (1).

*C. elegans lifespan determination*

Using the same worm strain (SS104), defined medium and *E. coli* OP50 and preparation method as for the healthspan assays, 25 sterile L4 worms were added to each Petri dish using up to 9 petri dishes per condition (225 worms). After 7 and 14 plates worms were transferred to fresh plates. From day 7 of adulthood onwards worms were scored for survival using standard techniques three times per week as described (2). Statistical significance was determined using Log Rank and Wilcoxon tests of the Kaplan–Meier survival model.

*Effects of substances on C. elegans gst-4 expression and resistance to paraquat*

Synchronised *C. elegans* containing a *gst-4p*:GFP::NLS transcriptional fusion transgene (CL2166) (3) were prepared from an egg lay by gravid adults at 20°C. After 48 hours, the population of L3/L4 progeny were transferred onto agar petri dishes containing either bifenazate or bromoxynil at concentrations of 50µM, 100µM, and 200µM, or DMSO only control). After a 24-hour exposure on compound plates, a sample of the early adult worms (n>15) were kept for imaging. The rest of the worms were rinsed off with M9 and treated with 150 mM paraquat in liquid for 2 hours. These worms were then washed with M9 and transferred back onto agar petri dishes. The ratio of alive and dead adult worms were assessed at this stage (n>90). Subsequently, live worms (n>50) for each condition were transferred onto fresh petri dishes (no compound) and assessed for survival 24 hours later. Imaging (around 28 hours after compound exposures) was achieved with a Leica MZ10F stereomicroscope with a GFP LP filter (LEICA 10450589) and illuminated by a CoolLED pE-300^white^, and an AmScope MD500 camera.

*Assessing hsp-6 expression in response to substances*

C. elegans containing the *hsp-6p*:GFP transcriptional fusion (SJ4100 *zcIs13(hsp-6p::GFP*)) (4) were added at the L4 stage to agar petri dishes containing bifenazate at 31.6 µM or 158 µM or bromoxynil at 100 µM or 500 µM. Worms were then compared by eye to the controls using a epifluorescent stereomicroscope after 2-3 hours and after 3 and 4 days with incubation at 24°C. No consistent differences were observed.

**Supplementary Tables:**

**Table S1. Multiple testing correction of survival comparisons using Bonferroni and Benjamini–Hochberg (BH) procedures.**

A total of 168 survival comparisons (2 species × 2 sexes × 15 substances × 3–4 dose groups) were analysed. The table lists the 10 smallest p‑values in ascending order.

For each comparison, adjusted significance was evaluated using:
(i) Bonferroni correction, controlling the family‑wise error rate, and
(ii) Benjamini–Hochberg (BH) procedure, controlling the false discovery rate (FDR).

The BH adjusted values (q‑values) were calculated from ranked p‑values and are shown after monotonic correction.

| **Substance, dose** | **Species, sex** | **p‑value** | **Rank (ascending p‑value)** | **Total tests (m)** | **BH critical value (p × m / rank)** | **BH adjusted q‑value (monotonic)** | **Significant (q ≤ 0.10)** | **Significant (q ≤ 0.20)** |
| --- | --- | --- | --- | --- | --- | --- | --- | --- |
| Clothianidin, 3000 | Rat,  male | 0.0006 | 1 | 168 | 0.1008 | 0.09576 | TRUE | TRUE |
| Bromoxynil,  600 | Rat, female | 0.00114 | 2 | 168 | 0.09576 | 0.09576 | TRUE | TRUE |
| Clothianidin, 3000 | Rat, female | 0.0024 | 3 | 168 | 0.1344 | 0.1008 | FALSE | TRUE |
| Cyproconazole, 100 | Mouse, female | 0.00258 | 4 | 168 | 0.10836 | 0.1008 | FALSE | TRUE |
| Bromuconazole, 100 | Mouse, male | 0.003 | 5 | 168 | 0.1008 | 0.1008 | FALSE | TRUE |
| Chlortoluron,  100 | Rat, female | 0.0055 | 6 | 168 | 0.154 | 0.1536 | FALSE | TRUE |
| Bromoxynil,  190 | Rat, female | 0.0064 | 7 | 168 | 0.1536 | 0.1536 | FALSE | TRUE |
| Cyproconazole, 200 | Mouse, male | 0.00797 | 8 | 168 | 0.16737 | 0.16737 | FALSE | TRUE |
| Ascorbic acid, 50000 | Mouse, male | 0.01 | 9 | 168 | 0.18667 | 0.18667 | FALSE | TRUE |
| Alanycarb,  300 | Mouse, male | 0.0192 | 10 | 168 | 0.32256 | 0.32256 | FALSE | FALSE |

Statistical notes:
*m* denotes the total number of comparisons (m = 168).
The Bonferroni correction adjusts the significance threshold as α / m (with α = 0.05).
The Benjamini–Hochberg (BH) procedure controls the expected false discovery rate (FDR) by comparing each ranked p‑value to its critical value (rank/m × α).
The q‑value represents the FDR‑adjusted p‑value after monotonic correction.

**Table S2. Toxicity summary for 15 substances that improve rodent survival in long-term studies.** Data obtained from the BfR Archive of Studies and Assessments. Numbers indicate either substance concentrations in food (ppm) or doses directly applied by oral gavage (in mg/kg bw per day). NOAEL: No Observed Adverse Effect Level, LOAEL: Lowest Dose with Observed Adverse Effect, MoA: Mode of action. Current classification obtained from either the website of European Chemicals Agency (ECHA, <https://www.echa.europa.eu/information-on-chemicals>, retrieved in December, 2024) or from proposals of the European Food Safety Authority (EFSA).

| **Substance / Toxicological endpoint** | **Genotoxicity** | **Reproductive toxicity (incl. developmental effects)** | **Neurotoxicity** | **Carcinogenicity** | **Chronic (organ) toxicity** | **Toxicity classification (oral route*)** |
| --- | --- | --- | --- | --- | --- | --- |
| **Alanycarb** | Negative | Reduced pup weight from 120 ppm onwards and lower pup survival at 480 ppm in a multi-generation study, NOAEL 30 ppm (rats) | Signs of cholinesterase inhibition at toxic doses | No indication | Nervous system (cholinesterases↓), liver (organ weight↑), blood (some evidence of anaemia); NOAELs 30 ppm in rats, 100 ppm in mice | Not listed by ECHA, no proposals by EFSA |
| **Ascorbic acid** | Negative | No indication | No indication | No indication | No indications up to highest tested doses of ca 2000 mg/kg bw per day (based on old studies in rats and mice) | None |
| **Bifenazate** | Negative | Lower body weight and some delay in female sexual maturation in a two-generation study on rats, NOAEL 80 ppm; variations in development of vascular system in rat foetuses, NOAEL 100 mg/kg bw per day | Weak evidence; NOAEL 13 mg/kg bw per day, based on reduced rearing at 35 mg/kg bw per day (rat) | No indication | Spleen, kidney and liver findings (organ weights, histopathology), anaemia; NOAELs 20 ppm in rats, 10 ppm in mice | STOT RE 2, H373 |
| **Bromoxynil** | Negative | Reduced bodyweight gain in offspring in a multi-generation study in rats, NOAEL 50 ppm; fetal malformations at a high (maternally toxic) dose of 40 mg/kg bw per day (rats) | No indication | Hepatocellular adenoma and carcinoma in male mice at 20-30 ppm and above and at 300 ppm in females but not relevant for humans due to MoA | Liver (organ weight↑, centrilobular hypertrophy, spongiosis hepatis), thyroid (reduced hormone levels, histopathology); NOAELs 60 ppm in rats, 30 ppm in mice | Repr. 2, H61d; Acute Tox. 3, H301 |
| **Bromuconazole** | Negative | Lower body weight gain in offspring, NOAEL 20 ppm (rat); increase in placental weight, ossification delay and skeletal variations in rat foetuses, NOAEL 10 mg/kg bw per day | No indication | Hepatocellular carcinoma at top dose levels of 2000 or 3000 ppm in rats and mice, probably not relevant to humans | Liver (organ weight↑, histopathology, clinical chemistry), adrenals (vacuolation); NOAELs 20 ppm (both rat and mouse) | Not listed by ECHA; EFSA proposal: Acute Tox. 4, H302; Repr. 2, H361d |
| **Chlorfenapyr** | Negative | Reduced pup survival in rats during postnatal days 1-4; tentative NOAEL 60 ppm | Vacuolation of white matter in the brain, spinal cord and optic nerve; NOAEL 60 ppm (rat) | No indication | Liver weight↑, red blood cell parameters↓; NOAELs 60 ppm in rats, 20 ppm in mice | Acute Tox. 4, H302 |
| **Chlorotoluron** | Negative | Resorption rate↑ and some evidence of skeletal anomalies in rabbits, NOAEL 50 mg/kg bw per day; lower number of implantation sites in dams in a two-generation study in rats, NOAEL 1000 ppm | No indication | Kidney tumours↑ and positive trend for liver tumours in male mice at 2500 ppm, NOAEL 500 ppm | Kidney weight↓, some evidence of anaemia; NOAEL 100 ppm (both in rats and mice) | Carc. 2, H351; Repr. 2, H361d |
| **Clothianidin** | Mixed results in vitro but overall negative (based on in vivo studies) | Low sperm motility in parental males, delayed sexual maturation in offspring in a two-generation study in rats, NOAEL 150 ppm; visceral anomalies and reduced ossification in rabbit foetuses, NOAEL 25 mg/kg bw per day | Limited evidence of reduced activity and neuro-developmental effects, perhaps due to systemic toxicity | No indication | Kidney (hyperplasia, mineralisation), liver (eosinophilic foci and hypertrophy), ovaries (interstitial cell hyperplasia), myocardial degeneration; NOAELs 150 ppm (rat), 350 ppm (mouse) | Acute Tox. 4, H302 |
| **Cyantraniliprole** | Negative | No indication | No indication | No indication | Liver (organ weight↑, histopathology, clinical chemistry); NOAELs 200 pm in rats, 150 ppm in mice | None |
| **Cyproconazole** | Negative | Malformations in rat and rabbit foetuses; NOAELs 2 mg/kg bw per day (rabbit), 12 mg/kg bw per day (rat) | No indication | Liver cell adenomas and carcinomas in male mice, human relevance equivocal; NOAEL 15 ppm | Liver (histopathology, clinical chemistry, organ weight↑); NOAELs 50 ppm in rats, 15 ppm in mice | Repr. 1B, H360D; STOT RE2, H373; Acute Tox. 3, H301; additional proposal by EFSA: Carc. 2, H351 |
| **Cyprodinil** | Negative | No indication | No indication | No indication | Liver (organ weight increase, histopathology), pancreas (hyperplasia of acinar cells); NOAEL 75 ppm in rats, 150 ppm in mice | None |
| **Dimethenamid** | Negative | Delayed ossification in rat foetuses, NOAEL 25 mg/kg bw per day | No indication | Equivocal increase in ovarian hyperplasia/adenoma in rats, NOAEL 700 ppm | Liver (organ weight↑ and histopathology); NOAELs 100 ppm (rat), 300 ppm (mouse) | Acute Tox. 4, H302 |
| **Etoxazole** | Negative | Increase in peri-/postnatal mortality of pups in a two-generation study in rats; NOAEL 400 ppm | No indication | No indication | Liver (organ weight↑ and histopathology), effects in teeth and bone suggestive of fluorosis; NOAELs 50 ppm (rat), 2250 ppm (mouse) | None |
| **Fenpyroximate** | Negative | No indication | No indication | No indication | Ovarian atrophy in mice; NOAELs (based on reductions in body weight and food consumption) 25 ppm in rats, 100 ppm in mice | Acute Tox. 3, H301 |
| **Lenacil** | Negative | Skeletal variations in rat foetuses↑ suggestive of developmental delay; NOAEL 100 mg/kg bw per day | No indication | Increase in mammary gland and uterine adenocarcinoma in female rats, NOAEL 250 ppm; liver adenoma and lung carcinoma in male mice, NOAEL:2500 ppm | Kidney, liver, spleen, and thyroid (organ weights↑, histopathology, clinical chemistry); NOAELs 250 ppm (rat) or 100 ppm (mouse) | Carc. 2, H351 |

*Any classification for endpoints such as acute dermal or inhalation toxicity, skin and eye irritation or skin sensitization was not taken into consideration for this table.

| **Substance** | **Class** | **Mode of action** | **Ref** | **Our data** | | | |
| --- | --- | --- | --- | --- | --- | --- | --- |
|  |  |  |  | **Intact cell OCR (LD20)** | **Intact cell OCR (LD80)** | **complex I-linked OCR, LD80** | **complex II-linked OCR, LD80** |
| Alanycarb | Insecticide | Cholinesterase activity inhibitor | 5 |  |  |  |  |
| Ascorbic acid | Fungicide, bacteriocide | Phytoalexin synthesis activator, water-soluble anti- or pro-oxidant | 6 |  |  |  |  |
| Bifenazate | Insecticide | GABA receptor agonist; ETC inhibitor | 7 | **↓** | **↓** | **↓** |  |
| Bromoxynil | Herbicide | Photosynthetic ETC inhibitor; OXPHOS uncoupler | 8 | **↑** | **↑** | **↑** | **↑** |
| Bromuconazole | Fungicide | Ergosterol biosynthesis inhibitor; Fungal CYP inhibitor | 9 |  | **↓** | **↓** |  |
| Chlorfenapyr | Pro-insecticide | Pro-drug to the active uncoupler, AC 303,268 (tralopyril) | 10 |  | **↑** |  |  |
| Chlortoluron | Fungicide | Photosynthetic ETC inhibitor | 11,12 |  |  | **↓** |  |
| Clothianidin | Insecticide | Targets AChR, suppresses NF-kB signalling in the honey bee | 13 |  |  |  |  |
| Cyantraniliprole | Insecticide | Disrupts the Ca^2+^ balance; Second generation ryanodine receptor | 14 |  | **↓** |  |  |
| Cyproconazole | Fungicide | Cytochrome P450 inhibitor (CYP51) | 15 |  |  |  |  |
| Cyprodinil | Fungicide | Methionine biosynthesis inhibitor; COX2 -PDG2 inhibitor | 16 |  |  | **↓** |  |
| Dimethenamid | Herbicide | Selective fatty acid synthesis inhibitor | 17 |  |  |  |  |
| Etoxazole | Insecticide | Inhibits chitin synthase 1, may enhance reactive oxygen species production | 18,19 |  | **↓** | **↓** | **↓** |
| Fenpyroximate | Acaricide | Blocks complex I by binding to ND5 | 20,21 | **↓** | **↓** | **↓** | **↓** |
| Lenacil | Herbicide | Inhibits photosynthesis (photosystem II) | 22 |  | **↓** |  |  |

**Table S3. Possible mechanisms of action of 15 substances as per literature and summary of their mitochondrial effects in human fibroblasts.** Red colour indicates that according to the literature, effects on electron transfer systems are indicated, albeit not necessarily in mammalian systems. Arrows indicate a significant effect of the substance in human fibroblasts. In mitochondrial OCR, where ‘**↓’** is shown, ‘complex I-linked’ indicates the inhibitory effects at complex I (i.e., the effects observed when used complex-linked substrate, pyruvate and malate), whereas that for ‘complex II-linked’ are those other than at complex I (i.e., when used complex II-linked substrate, succinate). OCR, Oxygen Consumption Rates; ETC, electron transport chain; OXPHOS, oxidative phosphorylation.

**Supplementary Figures:**

**Figure S1. Screening strategy.** ^1^Significance established by Cox test, Tarone test, or LogRank test.

**Figure S2. Example survival curves for rats and mice treated with bromoxynil.** A) female rats, B) male rats, C) female mice, D) male mice. P values are given when significant according to Gehan-Breslow test and Holm-Sidak pairwise comparisons.

**Figure S3. Human lung fibroblasts (MRC5) viability following 48h treatment with 15 selected substances at the indicated concentrations.** The maximum concentration tested for clothianidin, cyantraniliprol, dimethenamid, fenpyroximate and lenacil was 50 µM due to their poor solubility.

**Figure S4. Effects of 15 substances on complex I- and II- linked mitochondrial oxygen consumption rates (OCR) in permeabilised human lung fibroblasts. A)** Sequential measurements of mitochondrial OCR in the presence of complex I-linked substrates (Pyruvate 10mM and Malate 1mM) at basal, at State 3 (ADP 4mM), with substances at LD80 (see suppl. Fig. S2), with complex I inhibitor rotenone (5µM), and with the complex II substrate, succinate (4mM) added at the times indicated. **B)** % mitochondrial OCR at state 3 in the presence of complex I-linked substrate, with substances (at LD80) relative to that in the absence. **C)** % mitochondrial OCR at state 3 in the presence of complex II-linked substrate succinate, with substances (at LD80), relative to that in the absence (normalised to complex I-linked OCR, i.e. the OCR after ADP addition). Data from 3-14 measurements in 2-3 independent experiments using Seahorse XF Analyzer. One way ANOVA, differences to CON indicated by asterisks with * P<0.01, ***P<0.001, ****P<0.0001.

**Figure S5. Mitochondrial OCR (at state 4) in permeabilised MRC5 cells, supplemented with complex I-linked substrates 10mM Pyruvate and 1mM Malate in the presence of 5μM oligomycin.** At each arrow, mitochondrial uncouplers BAM15, FCCP or Niclosamide at 1μM (final concentration after 4 additions = 4μM), or bromoxinil or chlorfenapyr at 10μM (final concentration after 4 additions = 40μM) were added.

A	B

**Figure S6. *C. elegans* lifespan under bromoxynil or bifenazate treatments.** Bromoxynil treatment at 100 µM resulted in a significant shortening of *C. elegans* lifespan relative to vehicle treated controls, whereas bifenazate at 31.6 µM increased lifespan but it was not reproducibly significant. A) Lifespan experiment 1 control (n=118, mean age= 22.3 days of adulthood), bromoxynil (n=97, age= 20.2, p<0.01), bifenazate (n=75, age= 24.1, p<0.05) B) Lifespan experiment 2 control (n=196, mean age= 18.7 days of adulthood), bromoxynil (n=223, age= 16.7, p<0.001), bifenazate (n=219, age= 19.0, not significant). Bromoxynil increased healthspan during the days 1 to 7 (Figure 1), which may have been through a hormetic effect, but shortened the lifespan for older worms. Lifespans may be modulated differently at other concentrations, or when the treatments were given age-specifically.

A

B	C

**Figure S7**. Response of *gst-4* to bifenazate and bromoxynil and protection from paraquat. *C. elegans* expressing a *gst-4p*::GFP fusion protein were exposed to bifenazate or bromoxynil at 50, 100, and 200 µM each. (A) *gst-4* expression. At all concentrations of bromoxynil, *gst-4* expression was increased but not by any concentration of bifenazate. (B and C) Survival after 2 hours treatment with 150 mM paraquat. Both bifezanate and bromoxynil increased survival either immediately after exposure (B) or 24 hours later amongst the survivors (C). n>50 for each data point.

**Supplementary References:**

1	Zavagno, G., Raimundo, A., Kirby, A., Saunter, C. & Weinkove, D. Rapid measurement of ageing by automated monitoring of movement of C. elegans populations. *Geroscience* **46**, 2281-2293 (2024). [https://doi.org:10.1007/s11357-023-00998-w](rewritten://96d6eca2-dcf7-4d9a-9de4-03c120bb9801)

2	Virk, B., Correia, G., Dixon, D.P. et al. Excessive folate synthesis limits lifespan in the C. elegans: E. coliaging model. BMC Biol 10, 67 (2012).

3.	Link, C.D., Johnson, C.J.. Reporter Transgenes for Study of Oxidant Stress in Caenorhabditis elegans. Methods in Enzymology, Academic Press. **353**, 497-505. (2002), https://doi.org/10.1016/S0076-6879(02)53072-X.

4.	Yoneda, T., Benedetti, C., Urano, F., Clark, S.G., Harding, H.P., Ron, D. Compartment-specific perturbation of protein handling activates genes encoding mitochondrial chaperones. *J Cell Sci* **117** (18): 4055–4066 (2004). doi: <https://doi.org/10.1242/jcs.01275>

5	*CHEBI Onthology: 38463 - alanycarb*, <https://www.ebi.ac.uk/chebi/chebiOntology.do?treeView=true&chebiId=CHEBI:38463>

6	Massie, H. R., Aiello, V. R. & Doherty, T. J. Dietary vitamin C improves the survival of mice. *Gerontology* **30**, 371-375 (1984). [https://doi.org:10.1159/000212659](rewritten://3a6b5496-1895-4601-8f4b-3b871adf7a24)

7	Van Nieuwenhuyse, P., Van Leeuwen, T., Khajehali, J., Vanholme, B. & Tirry, L. Mutations in the mitochondrial cytochrome b of Tetranychus urticae Koch (Acari: Tetranychidae) confer cross-resistance between bifenazate and acequinocyl. *Pest Manag Sci* **65**, 404-412 (2009). [https://doi.org:10.1002/ps.1705](rewritten://6ee56efd-be87-4785-9fda-e78d5c9f3d08)

8	*US EPA Reristration Eligibility Decision: Bromoxynil*, https://archive.epa.gov/pesticides/reregistration/web/pdf/2070red.pdf

9	*Pesticide Properties Database: Bromuconazole (Ref: LS 860263)*, <https://sitem.herts.ac.uk/aeru/ppdb/en/Reports/97.htm>

10	*US EPA Pesticide Fact Sheet: Chlorfenapyr* https://www3.epa.gov/pesticides/chem_search/reg_actions/registration/fs_PC-129093_01-Jan-01.pdf

11	Broser, M. *et al.* Structural basis of cyanobacterial photosystem II Inhibition by the herbicide terbutryn. *J Biol Chem* **286**, 15964-15972 (2011). https://doi.org:10.1074/jbc.M110.215970

12	Diaz-Munoz, G., Miranda, I. L., Sartori, S. K., de Rezebde, D. C. & Diaz, M. A. N. in *Studies in Natural products Chemistry* Vol. 58 (ed Atta-ur-Rahman) 313-338 (Elsevier, 2018).

13	Di Prisco, G. *et al.* Neonicotinoid clothianidin adversely affects insect immunity and promotes replication of a viral pathogen in honey bees. *Proc Natl Acad Sci U S A* **110**, 18466-18471 (2013). [https://doi.org:10.1073/pnas.1314923110](rewritten://a4b30f72-6a5c-4f10-bd8f-e1ff04598d19)

14	Sparks, T. C. *et al.* Insecticides, biologics and nematicides: Updates to IRAC's mode of action classification - a tool for resistance management. *Pestic Biochem Physiol* **167**, 104587 (2020). [https://doi.org:10.1016/j.pestbp.2020.104587](rewritten://26abdfa8-d308-4901-a404-cd6d267e7e97)

15	He, Z. *et al.* Stereostructure-activity mechanism of cyproconazole by cytochrome P450 in rat liver microsomes: A combined experimental and computational study. *J Hazard Mater* **416**, 125764 (2021). [https://doi.org:10.1016/j.jhazmat.2021.125764](rewritten://64df00c7-0c77-4b8d-ae05-c22521097d59)

16	Kugathas, S. *et al.* Effects of Common Pesticides on Prostaglandin D2 (PGD2) Inhibition in SC5 Mouse Sertoli Cells, Evidence of Binding at the COX-2 Active Site, and Implications for Endocrine Disruption. *Environ Health Perspect* **124**, 452-459 (2016). [https://doi.org:10.1289/ehp.1409544](rewritten://22fff0c0-ee55-4eea-bde5-0eb1a466c412)

17	Lamberth, C. in *Bioactive Carboxylic Compound Classes* 293-302 (2016).

18	Demaeght, P. *et al.* High resolution genetic mapping uncovers chitin synthase-1 as the target-site of the structurally diverse mite growth inhibitors clofentezine, hexythiazox and etoxazole in Tetranychus urticae. *Insect Biochem Mol Biol* **51**, 52-61 (2014). [https://doi.org:10.1016/j.ibmb.2014.05.004](rewritten://83bf2598-ce6d-44e9-825b-3908109eec2b)

19	Sun, D., Pang, J., Fang, Q., Zhou, Z. & Jiao, B. Stereoselective toxicity of etoxazole to MCF-7 cells and its dissipation behavior in citrus and soil. *Environ Sci Pollut Res Int* **23**, 24731-24738 (2016). <https://doi.org:10.1007/s11356-016-7393-7>

20	Boerner, H., Schlueter, K. & Aumann, J. *Pflanzenkrankheiten und Pflanzenschutz*. 8 edn, (Springer, 2009).

21	Shiraishi, Y., Murai, M., Sakiyama, N., Ifuku, K. & Miyoshi, H. Fenpyroximate binds to the interface between PSST and 49 kDa subunits in mitochondrial NADH-ubiquinone oxidoreductase. *Biochemistry* **51**, 1953-1963 (2012). [https://doi.org:10.1021/bi300047h](rewritten://afdc23c1-f86f-4453-b92e-f533c3e33de4)

22	*PesticideProperties Database: Lenacil (Ref: DPX B634)*, <https://aeru.herts.ac.uk/aeru/ppdb/en/Reports/417.htm>
